# Supplementary material for: Self-Monitoring Diabetes-Related Foot Ulcers with the MyFootCare App: A Mixed Methods Study
Source: Sensors (Basel). 2023 Feb 24;23(5):2547. doi: 10.3390/s23052547 (PMC10006972; doi:10.3390/s23052547)
Supplement: Supplementary file 1 [file sensors-23-02547-s001.zip › sensors-2088821-supplementary.pdf]

Guide for Patient and Carer Interview 1 (90 mins)

Introduction (5min)

- ☐ **Welcome & thank** for support & time
- ☐ **Introduce myself**

- ☐ **Project**
  - ☐ Aims to **support people with DFUs and caregivers in care at home**
  - ☐ **MyFootCare app**: track healing progress & visualise personal goals
  - ☐ **Study aims**: understand app use in daily life 3 months
    improve usability (easy to use),
    usefulness for self-care and engagement with podiatrist

- ☐ **Interview today**: learn about your self-care practices, care support, mobile phone use; show you MyFootCare app and provide you with a phone with the app installed on it
  - ☐ **No right or wrong answers; no** stupid questions,
  - ☐ Give your **honest & critical feedback**
  - ☐ **Completely voluntary, right to withdraw at any time**

- ☐ **Have you got any questions about consent form?**
- ☐ Which **voucher?**

- ☐ **<u>Start audio recorder</u>**

- ☐ May **take notes,** so I can go back to our conversation later when I analyse the data

Foot ulcer and self-care (use cards, 15 mins)

1) **How long** have you had the **foot ulcer** for & how did you get it?
    a. Keep follow-up discussion to last year

2) What is your **aim?** What would you like the foot to be in 6 months?

3) I'd like to discuss what you do **at home to care for your foot and ulcer.** To discuss this, we use **cards.** Which cards do you identify with the most?
    a. Change dressings
    b. Regular checking of the foot
    c. Wear offloading device
    d. Everyday hygiene
    e. Wear footwear
    f. Doctor and podiatrist interactions
    g. Identify infection
    h. Caring for the other foot
    i. Tracking progress

Diabetes and overall health (5 mins)

4) **How long** have you had **diabetes** for?
(when/how was it diagnosed?)

5) How do you **manage your diabetes:** diet, medication, sugar levels, exercise? Can you talk me through what you did yesterday?

6) When was the **last time you got your eyes and kidney checked?**

7) Very briefly, have you got any **other health concerns?**

Everyday life (cards, 10 mins)

8) Pick 3-5 most relevant cards for you to talk about how the ulcer and self-care affects **everyday life**

a. **Relationship** with other family and friends
b. **Work:** Paid employment, household work, yard work
c. Health system
d. Activities and hobbies
e. Driving and transport
f. Fatigue and wellbeing

Smartphone use (5 mins)

9) **What phone do you (or your partner) have?**
Do you have an Apple iPhone/Samsung/Android?

10) What are your **3 favourite applications?**
Could you show us one of these apps on your phone?
How do they work?
How often do you use it (daily/weekly/less frequent)?
What do you like about the app?

11) Do you use your **phone to take photos**? What photos do you take?

12) Is there a **photo that means a lot to you?**
Could you **show it to me** and tell me more about what it means to you?

13) Have you ever **taken photos of your foot ulcer**?
Tell me more about the photo. When? Why? Who took the photo? Who saw them? Would you be interested in seeing photos of your ulcer to see its healing progress?

Short break needed?

MyFootCare walkthrough (20 mins)

MyFootCare intro:

- **created with patients and health professionals** to help you in your ulcer self-care.
- **3 key features:** (1) **personal goals**,
  (2) **reminders** to conduct self-care,
  (3) **feedback** about the progress of your ulcer and photos of your feet.

Together, we will now **go through app** and **cheat sheet to ensure you are confident** in using MyFootCare

- Want your **honest feedback** today and from trying it out over the next few weeks
- **Think out loud** as we move through the different features of the app
- What do you think about a **feature**?
- What **questions** do you have about it?

14) Find MyFootCare **app**

15) MyFootCare Home screen & motivational image

- **What motivates you in your life?**
  Can you tell us more about it?
- Do you have an **image** on your own phone or study phone that reflects that motivation?
  - Take photo of photo (landscape)
  - Select from photos provided
  - Do later at home

16) **Take a foot photo** --> see cheat sheet

- What do you think about the photo taking?
- Do you have a **person to help you?**
- How can we make it easier for you?
- What questions do you have about it?

17) **Check wound size**
> Use image taken during consultation

- Assistance – **stylus?**
- **Notes:** Do you keep any notes about your foot ulcer (in writing, in your head)?
  Anything that your podiatrist asks to note down?
-

18) **Review graph & 4 week goal**

- International guidelines suggest that the ulcer should be 50% improved within 4 weeks.
  If you are tracking close to it, then it is a good outcome. However, if you are not, then this might be something to discuss with your clinician.
- **How realistic do you think this goal is for you?**
- What factors do you think may make it likely or unlikely to reach that goal?
-

19) **Review foot photos**

- Are you **interested in seeing photos of your foot?** What interests you?
- **Who** would you show these images to?
-

20) **Set reminder**

- When was the last time you changed dressings?
  Do you have a routine for changing dressing
  (when, how often)?
  When is a good time for a reminder and how often?
  go to :. Notifications - Set time and frequency
-

21) **Looking after the phone**

- Charge
- keep clean -wipes
- look well after it
- bring to next interview and consultation – observe

MyFootCare Evaluation (10 mins)

22) Can you **rate how useful the different features** of the application are for you? On a scale from 1 to 10, where 1 is not useful and 10 is very useful, how would you rate the following features:

- **Motivational Image**

  1 2 3 4 5 6 7 8 9 10

- **Reminder notifications** to care for foot

  1 2 3 4 5 6 7 8 9 10

- **Tracking progress** of the wound

  1 2 3 4 5 6 7 8 9 10

- **See photos of wound**:

  1 2 3 4 5 6 7 8 9 10

- **Sharing data with podiatrist**

  1 2 3 4 5 6 7 8 9 10

- **MyFootCare app overall**

  1 2 3 4 5 6 7 8 9 10

23) With reference to the previous question, can you please tell us **why** you gave these ratings?

24) Which three things would you change to improve the application?
Probe more: What could be improved? And What do you miss?

1.
2.
3.

25) When we make changes to the application, **which three things should we keep from the current application?**

1.
2.
3.

Closing and next steps (5 mins)

26) Do you have any questions or comments about this project or the app?

☐ **Stop recording**

☐ **next steps:**

- **use app up to 3 months** <u>every time you change wound dressing</u>
- **follow up phone call** in a few days to help with any questions you might have
- don't hesitate to **contact us** if you have questions at any time about the app/study

☐ **make appointment in in 3 weeks**:

- **bring phone** with data and photos to **consultation** and discuss with podiatrist – we want to observe if and how the data gets used during consultation
- **interview afterwards** to discuss MyFootCare use and progress with DFU

☐ **Thank** for participation

Guide for Interview 2 (45 mins)

Introduction (2 mins)

- ☐ **Welcome & thank** for support & time
- ☐ **Talk about**
  - o **Progress**
  - o **Experience with MyFootCare App**
- ☐ **Start audio recorder**

- ☐ May **take notes,** so I can go back to our conversation later when I analyse the data

Progress (10 mins)

27) In your own words, **how do you think your foot ulcer has progressed in the last 3 months, since the start of the study?**
has the foot ulcer:
   a. Better or worse?
   b. Healed?
   c. Bigger or smaller?
   d. Deeper or shallower?
   e. Tissue quality / less or more callused
   f. Complications: infections, admission to hospital, on antibiotics?

28) Do you remember what your **aim was at the start of the study – has it changed?**

MyFootCare walkthrough (15 mins)

Start screen recording software
Open the app – talk us through the data

Graph, Photos, Notes

Talk us through graph, photos, notes

29) Let's look at the **progress graph** on the app. What do you see there?
    any data?
    current %
    reached 4 week goal?
    any ups, or downs?
    how many foot checks?

30) Outliers: show **unusual (high or low) foot checks?**

31) Periods of intense or scare use (according to graph) – what happened?
    What factors may have influenced your engagement?

Photo taking and analysis

32) Could you please **analyse photo taken during consultation and talk out loud what you think?**

33) When was the **last photo/foot check taken?**
- Date?
- What progress %?
- improved or worse?

34) Can you **open the photo** (click on orange dot in graph) and **tell us more about it?**
- What do you see on the photo?
- How does the wound look like?
- Anything *interesting or surprising* beyond the %?
  (e.g., redness, healing, depth, …)

35) What is the **quality of the last photo?**
    In focus?
- Entire foot visible within the outline
- Straight on (camera parallel to the foot)
- Minimize skin (e.g., ankle, leg) in the background

36) Interested in the **process of taking photos**, aware that it can be difficult. Can you tell us more about how the (last) photo was taken?
- ***Who*** took the photo?
- *Where, and when?*
- Any other *support* (selfie stick, tripod)?
- Challenges or questions?
- Suggestions for improvement?

37) Process of analysing photos
- *When: immediately* following on after taking the photo, *or later on*?
- *Who analysed photo*
- Challenges or questions?
- Suggestions for improvement?

38) Looking at the **photo and the green lines**, **how accurately did the app recognize the foot and the wound?**
Foot - green line accurate or any additions or omissions?
Ulcer - green line accurate or any additions or omissions?

Photo progress and accuracy

39) **Compare last photo with first photo?** What difference do you see?

40) Over the 4 weeks, do you feel the **MyFootCare app was accurately tracking your foot ulcer?**
Why (not)?
Show us examples on phone

41) Did you add notes to photos?
yes / no
What did you note?
Show example & tell us more

Motivational image

42) Can you **open the app** and show us the home screen. **What motivational image is on the top?**
- pre-loaded or uploaded personal photo?

Can you tell us what you like about the image?
What might be motivating about it?

☐ **create screen shot of home page**
- Press Power button and Volume down at same time
- Hold down until you hear a click or sound
- You will get a notification that your screenshot was captured, and that you can share or delete it.

43) Did you have **any other images before**?
can you show them to us?

Reminder notifications

44) Did you **notice any reminders?**
- updates to settings?
- Challenges or questions?
- Suggestions for improvement?

45) Have you **added any reminders yourself?**

46) What was the **impact of having 2 phones - any difference with your own phone?**

- For reminders
- Motivational image
- Taking photos
- General use/ carrying phone all the time

MyFootCare uptake & engagement (5 mins)

Usage patterns

47) When was the last time you opened the app? What for?

48) Did you use the **app yourself or did anyone else use the app with you,** e.g., to provide support with photos?

49) When was the last time the dressing came off?
- Did you use the app then to check size? YES NOW
- if YES – go to next question
- if NO, **what prevented you from using it?**

(reassure participant that it is fine not to use – we want to learn about MyFootCare in an honest manner, and we will discuss later how to make the app better)

Patient-clinician interaction during consultation (5 mins)

50) What did you think about sharing the app data during the consultation?

*51) Was it **beneficial** or not? Why/why not?*

52) Was it **helpful for the conversation**, learn about progress, ask questions, discuss self-care, life outside clinic, concerns, …?

53) Did you **learn anything new** about MyFootCare or your ulcer from discussing the app with your podiatrist?

54) Have you **shown your MyFootCare data to anyone else?** (partner, family, GP, other health professionals) What did you show and discuss?

55) Would you **discuss MyFootCare again during a consultation?** Why, why not?

56) Would you **recommend to other patients** using the app to prompt conversations about topics related to your health and wellbeing? Why, why not?

Sharing & Outcomes

57) Have you **discussed MyFootCare with anyone else?**
- with whom?
- When, what context?
- what did you discuss?

58) Have you **shown MyFootCare to anyone else?**
- with whom?
- When, what context?
- what did you discuss?

MyFootCare Usefulness Evaluation (5 mins)

59) Can you **rate how useful the different features** of the application are for you? On a scale from 1 to 10, where 1 is not useful and 10 is very useful, how would you rate the following features:

Can you please also **briefly tell us why** you give this rating?

- **MyFootCare app overall**

  1 2 3 4 5 6 7 8 9 10

- **Motivational Image**

  1 2 3 4 5 6 7 8 9 10

- **Reminder notifications** to care for foot

  1 2 3 4 5 6 7 8 9 10

- **Tracking progress** of the wound

  1 2 3 4 5 6 7 8 9 10

- **See photos of wound**:

  1 2 3 4 5 6 7 8 9 10

- **Sharing data with podiatrist**

  1 2 3 4 5 6 7 8 9 10

60) Did **MyFootCare provide any benefits to**

a. Support self-care
b. Track progress
c. Share / discuss with others
d. Feel motivated
e. Learn new things
f. …
g. YES – how? Example?
NO – why not?

Improvements & Future Use (5 mins)

61) Which three things would you change to improve the application for you?

Probe more: What could be improved? And What do you miss?

4.
5.
6.

62) What (else) would make you use MyFootCare more often in the future?

63) Will you use MyFootCare more often in the future?

YES, why?

NO, why?

Closing and next steps (3 mins)

64) Do you have **any questions or comments** about this project or the app?

☐ **Stop recording**

☐ **next steps:**

- **use app up to 2 months** every time you change wound dressing
- don’t hesitate to **contact us** if you have questions at any time about the app/study

☐ **make appointment in 2 months for final interview**

☐ **Thank** for participation

Interview 3 Guide (60mins)

Introduction (2 mins)

- ☐ **Welcome & thank** for support & time
- ☐ Aim: talk about progress of your ulcer, reflect on MyFootCare how you used it and whether it has been useful in any way for you
- ☐ **Voucher**
- ☐ Any questions before we start?
- ☐ **Start audio recorder**
- ☐ May **take notes,** so I can go back to our conversation later when I analyse the data

Progress (15 mins)

65) In your own words, **how do you think your foot ulcer has progressed in the last 3 months, since the start of the study?**
has the foot ulcer:

a. Better or worse?
b. Healed?
c. Bigger or smaller?
d. Deeper or shallower?
e. Tissue quality / less or more callused
f. Complications: infections, admission to hospital, on antibiotics?

66) Do you remember what your **aim was at the start of the study – has it changed?**

67) What has **contributed to your progress?**
To discuss this, we use **cards.** Any cards that are particularly relevant for your situation?
Can you put them in order of importance?
(take photo)
self-care

a. Change dressings
b. Regular checking of the foot
c. Wear offloading device
d. Everyday hygiene
e. Wear footwear
f. Doctor and podiatrist interactions
g. Identify infection
h. Caring for the other foot
i. Tracking progress

everyday life

j. **Relationship** with other family and friends
k. **Work:** Paid employment, household work, yard work
l. Health system
m. Activities and hobbies
n. Driving and transport
o. Fatigue and wellbeing

68) **What has made progress difficult?** What has held progress back?
(use cards)

69) Are you **satisfied** with your progress?

a. What are you satisfied with?
b. What would you have liked to achieve?

70) If you are unsatisfied, why?
what could have been done differently? **What would need to change?**

MyFootCare walkthrough (15 mins)
(set up screen recording with DU recorder)

Open the app – talk us through the data

Graph, Photos, Notes

Talk us through graph, photos, notes

71) Let's look at the **progress graph** on the app. What do you see there?
    any data?
    current %
    reached 4 week goal?
    any ups, or downs?
    how many foot checks?

72) Outliers: show **unusual (high or low) foot checks?**

73) Periods of intense or scare use (according to graph) – what happened?
    What factors may have influenced your engagement?

Photo taking and analysis

74) When was the **last photo/foot check taken?**
- Date?
- What progress %?
- improved or worse?

75) Can you **open the photo** (click on orange dot in graph) and **tell us more about it?**
- What do you see on the photo?
- How does the wound look like?
- Anything *interesting or surprising* beyond the %?
  (e.g., redness, healing, depth, …)

76) What is the **quality of the last photo?**
In focus?
- Entire foot visible within the outline
- Straight on (camera parallel to the foot)
- Minimize skin (e.g., ankle, leg) in the background

77) Interested in the **process of taking photos**, aware that it can be difficult. Can you tell us more about how the (last) photo was taken?
- ***Who*** took the photo?
- *Where, and when?*
- Any other *support* (selfie stick, tripod)?
- Challenges or questions?
- Suggestions for improvement?

78) Process of analysing photos
- *When: immediately* following on after taking the photo, *or later on*?
- *Who analysed photo*
- Challenges or questions?
- Suggestions for improvement?

79) Looking at the **photo and the green lines**, **how accurately did the app recognize the foot and the wound?**
Foot - green line accurate or any additions or omissions?
Ulcer - green line accurate or any additions or omissions?

Photo progress and accuracy

80) **Compare last photo with first photo?** What difference do you see?

81) Over the 12 weeks, do you feel the **MyFootCare app was accurately tracking your foot ulcer?**
Why (not)?
Show us examples on phone

82) Did you add notes to photos?
yes / no
What did you note?
Show example & tell us more

Motivational image

83) Can you **open the app** and show us the home screen. **What motivational image is on the top?**
- pre-loaded or uploaded personal photo?

Can you tell us what you like about the image?
What might be motivating about it?

☐ **create screen shot of home page**
- *Press Power button and Volume down at same time*
- *Hold down until you hear a click or sound*
- *You will get a notification that your screenshot was captured, and that you can share or delete it.*

84) Did you have **any other images before**?
can you show them to us?

Reminder notifications

85) Did you **notice any reminders?**
- updates to settings?
- Challenges or questions?
- Suggestions for improvement?

86) Have you **added any reminders yourself?**

87) What was the **impact of having 2 phones - any difference with your own phone?**
- For reminders
- Motivational image
- Taking photos
- General use/ carrying phone all the time

MyFootCare Usage patterns (5 mins)

88) When was the last time you opened the app? What for?

89) Did you use the **app yourself or did anyone else use the app with you,** e.g., to provide support with photos?

90) **How often** did you use MyFootCare during the last week? What for?

91) **What time of the day** did you usually open the app?
Related to dressing change?
Embedded in everyday life?

92) When was the last time the dressing came off?
- Did you use the app then to check size? YES NOW
- if YES – go to next question
- if NO, **what prevented you from using it?**
<u>(reassure participant that it is fine not to use – we want to learn about MyFootCare in an honest manner, and we will discuss later how to make the app better)</u>

93) Which **parts of the app did you use most/least?**
a. Progress
b.

94) Did you have **any difficulties using app?**

Sharing & Outcomes (5 mins)

95) Have you **discussed MyFootCare with anyone else?**
- with whom? (partner, family, GP, other health professionals)
- When, what context?
- what did you discuss?

*96) Was it **beneficial** or not? Why/why not?*
e.g., learn about progress, ask questions, discuss self-care, life outside clinic, concerns, …

97) Would you recommend to others discussing MyFootCare again during a consultation with GP, podiatrist, …?
Why, why not?

MyFootCare Usefulness Evaluation (5 mins)

98) Can you **rate how useful the different features** of the application are for you? On a scale from 1 to 10, where 1 is not useful and 10 is very useful, how would you rate the following features:

Can you please also **briefly tell us why** you give this rating?

- **MyFootCare app overall**

  1 2 3 4 5 6 7 8 9 10

- **Motivational Image**

  1 2 3 4 5 6 7 8 9 10

- **Reminder notifications** to care for foot

  1 2 3 4 5 6 7 8 9 10

- **Tracking progress** of the wound

  1 2 3 4 5 6 7 8 9 10

- **See photos of wound**:

  1 2 3 4 5 6 7 8 9 10

- **Sharing data with podiatrist**

  1 2 3 4 5 6 7 8 9 10

99) Did **MyFootCare provide any benefits to**

a. Support self-care
b. Track progress
c. Share / discuss with others
d. Feel motivated
e. Learn new things
f. …
g. YES – how? Example?
NO – why not?

-

Improvements & Future Use (5 mins)

100) Which three things would you change to improve the application for you?

Probe more: What could be improved? And What do you miss?

7.
8.
9.

101) Will you use MyFootCare more often in the future?

would you continue using on your own phone

YES, why?

NO, why?

102) What **questions** do you have about the app or the study?

- ☐ **Stop recording**
- ☐ **Thank** for participation

Cards used for card sorting during interviews

During the interview, participants were shown these cards and asked to choose the cards most relevant to their circumstances. The front of each card showed an image, and the back of each card showed questions related to the topic for discussion during the interview.

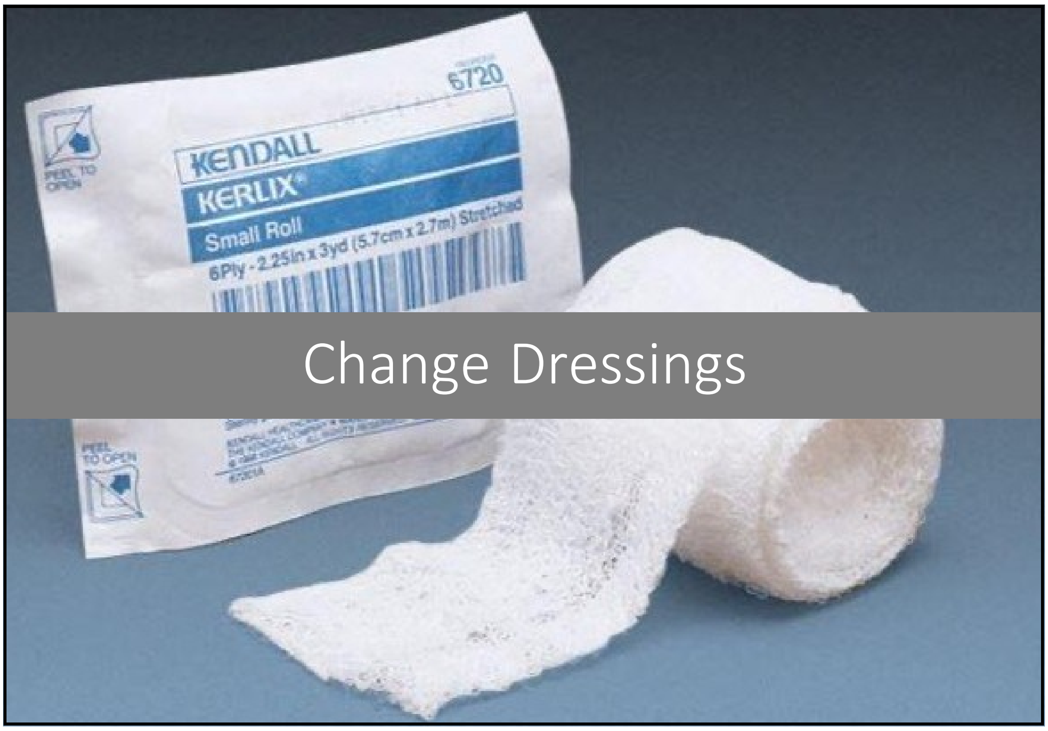

1

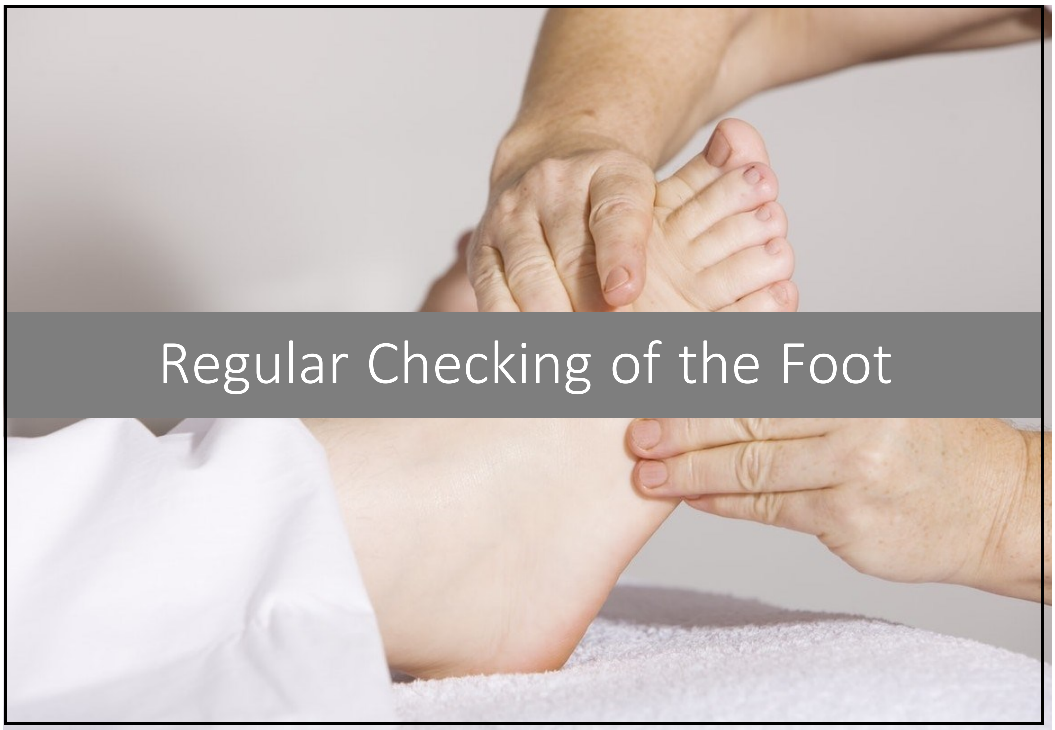

2

Change Dressings

Can you tell me more about the last time you changed your dressing at home?

Have you ever experienced difficulties with changing dressings? Can you give me an example?

Have you ever had a positive experience with changing dressings? Can you give me an example?

Does anyone help you with this activity? Who? How does s/he provide help?

3

Regular Checking of the Foot

How often do you check your foot?

Can you tell me about the last time you checked your foot?

Have you ever experienced difficulties with your foot? Can you give me an example?

Does anyone help you with this activity? Who? How does s/he provide help?

4

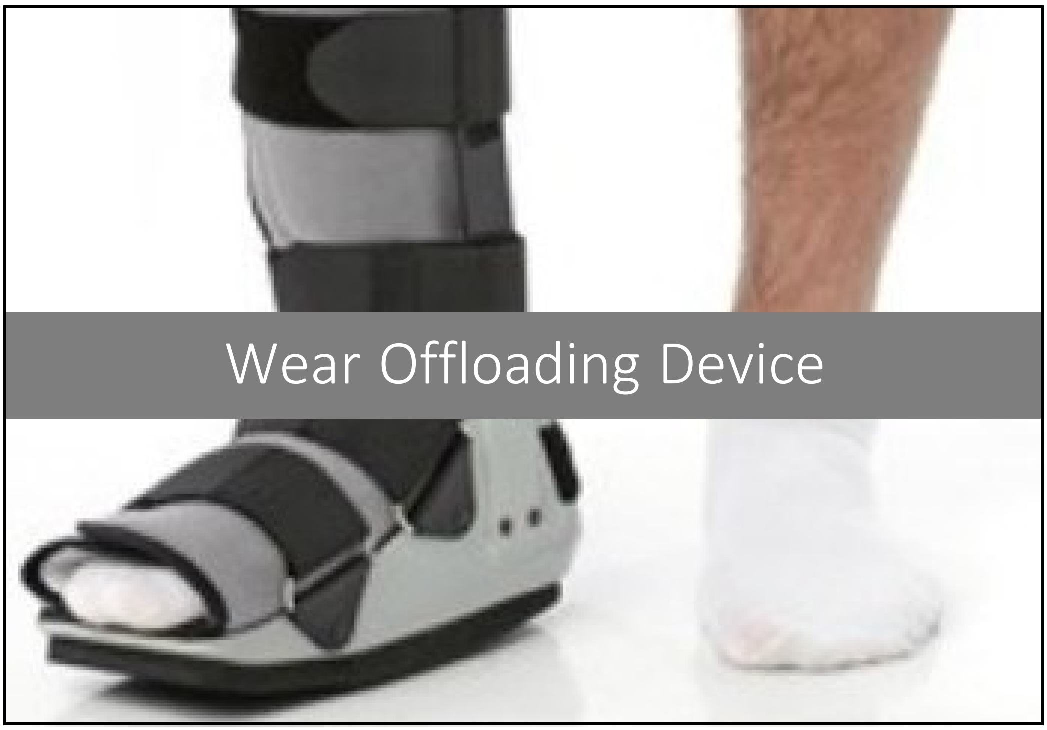

5

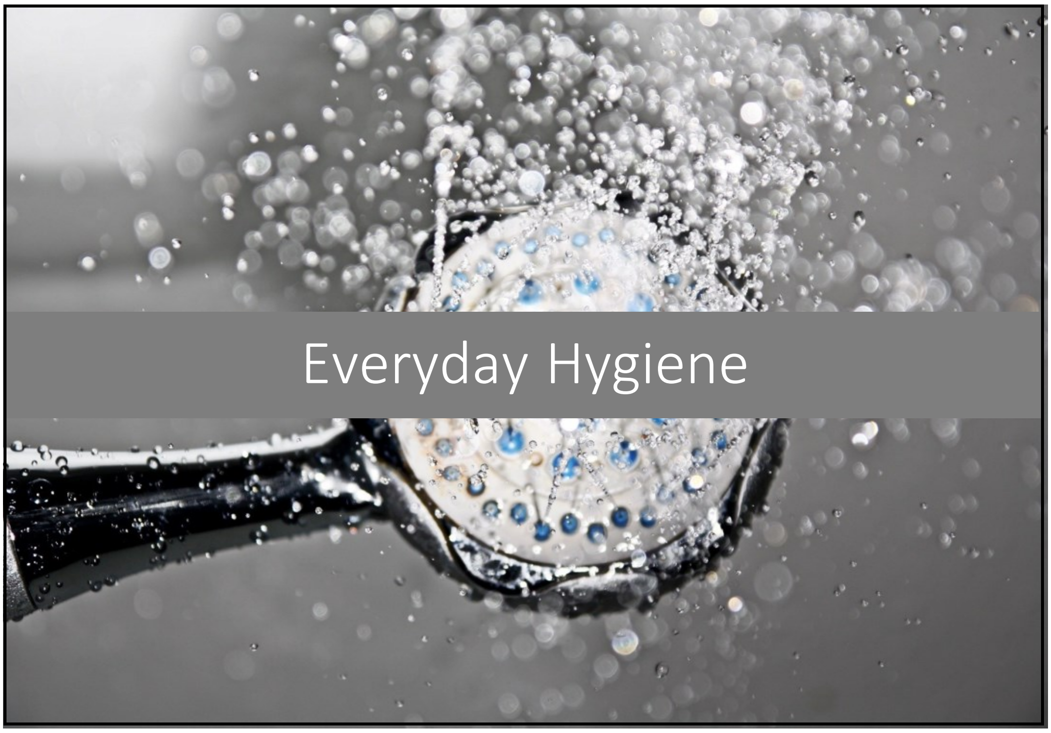

6

Wear Offloading Device

Can you tell me more about the last time you were wearing an offloading device at home?

When do you take your offloading device off?

Are there situations where you don’t wear the offloading device? What do you wear then?

Have you ever experienced difficulties with it? Can you give me an example?

Have you ever had a positive experience with it? Can you give me an example?

Does anyone help you with this? Who? How does s/he provide help?

7

Everyday Hygiene

How do you keep your foot ulcer clean?

How do you keep it dry in the shower?

Can you give me an example where it went really well?

Do you have an example where it didn’t go well?

Does anyone help you with this activity? Who? How does s/he provide help?

8

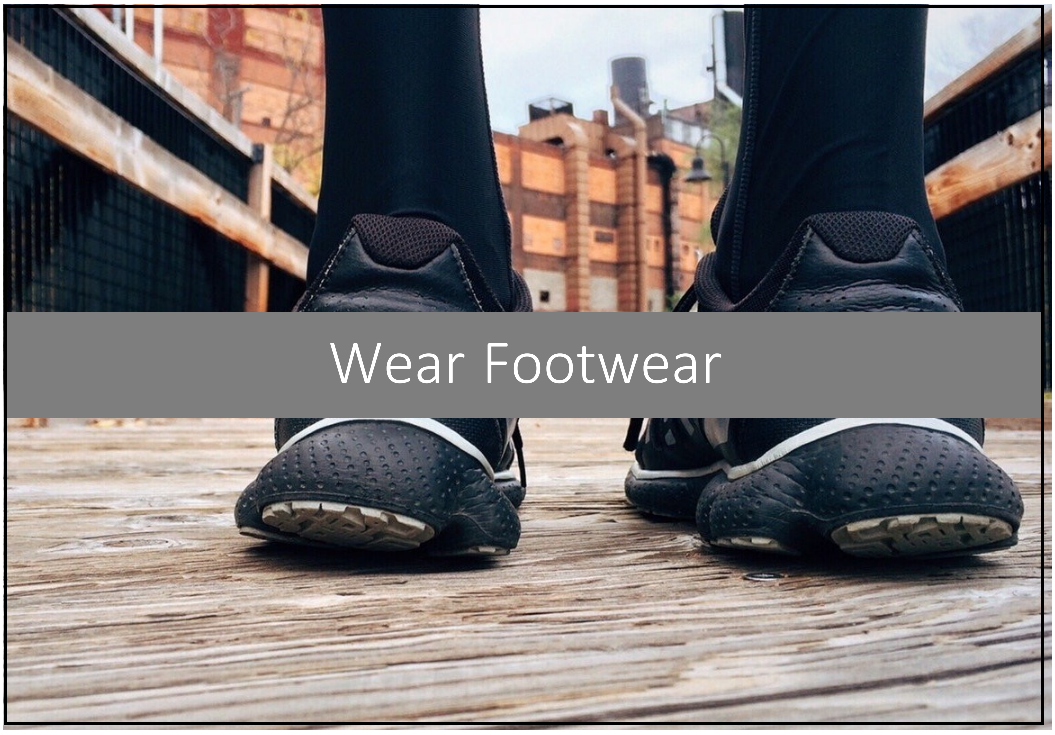

9

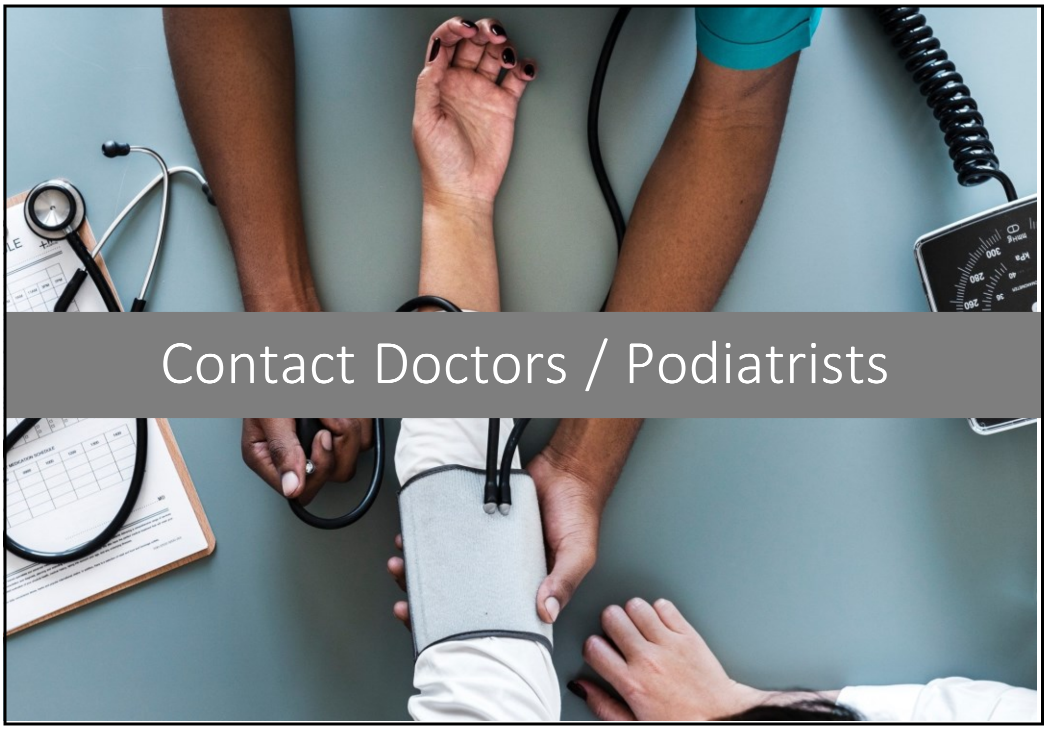

10

Wear Footwear

What do you wear on the good foot to protect the skin?

Can you tell me about the last time you bought new pair of shoes? How did your feet go?

Have you ever experienced difficulties with your footwear? Can you give me an example?

Have you ever had a positive experience with your footwear? Can you give me an example?

Does anyone help you with this activity? Who? How does s/he provide help?

11

Contact Doctors / Podiatrists

Can you tell us more about your doctor/podiatrist?

If you have a question for which you don't need an appointment, can you reach them by phone?

How often do you see them?

When was the last time you made an appointment on short-term notice? Why

What are their strengths?

12

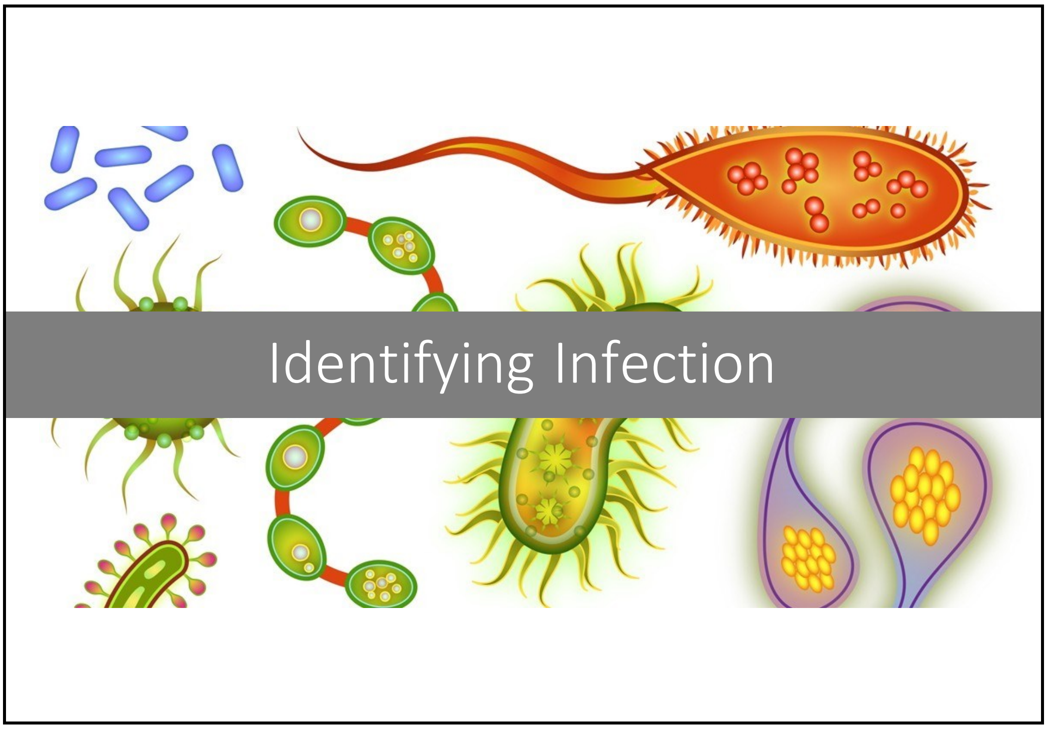

13

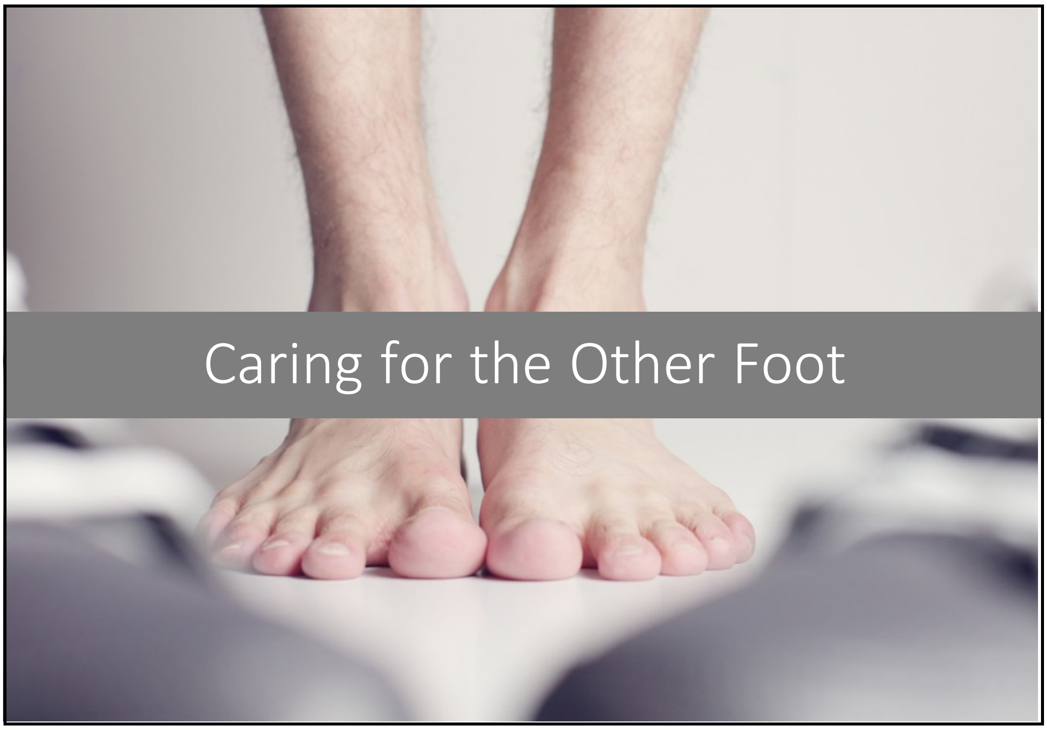

14

Identifying Infection

What do you do to identify infection?

What are the difficulties in identifying infection?

Does anyone help you with this activity? Who? How does s/he provide help?

Have you ever experienced an infection of the foot? How did you notice it? What did you do about it?

15

Caring for the other Foot

What do you do to care for the other foot?

Have you ever experienced difficulties the other foot?

Does anyone help you with this activity? Who? How does s/he provide help?

16

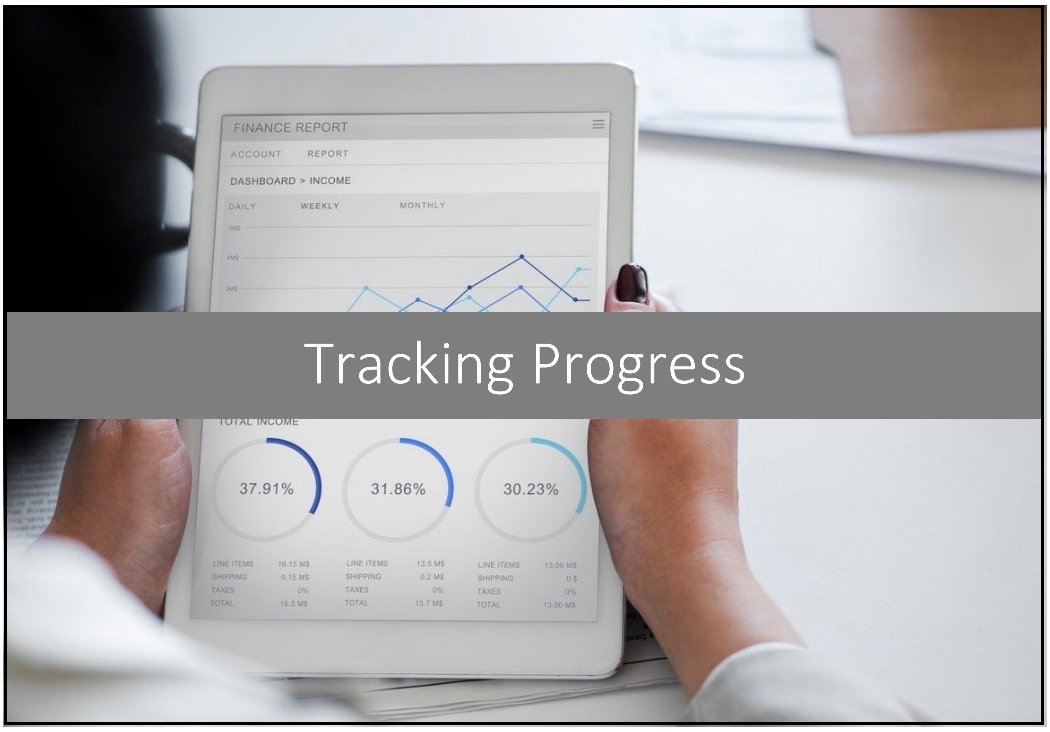
Tracking Progress
FINANCE REPORT
ACCOUNT
REPORT
DASHBOARD > INCOME
DAILY
WEEKLY
MONTHLY
37.91%
31.86%
30.23%

17

18

Tracking Progress

How do you notice progress with your foot ulcer?

Do you use any tools, notes, photos, apps, etc?

Does anyone help you with this activity? Who? How does s/he provide help?

19

20

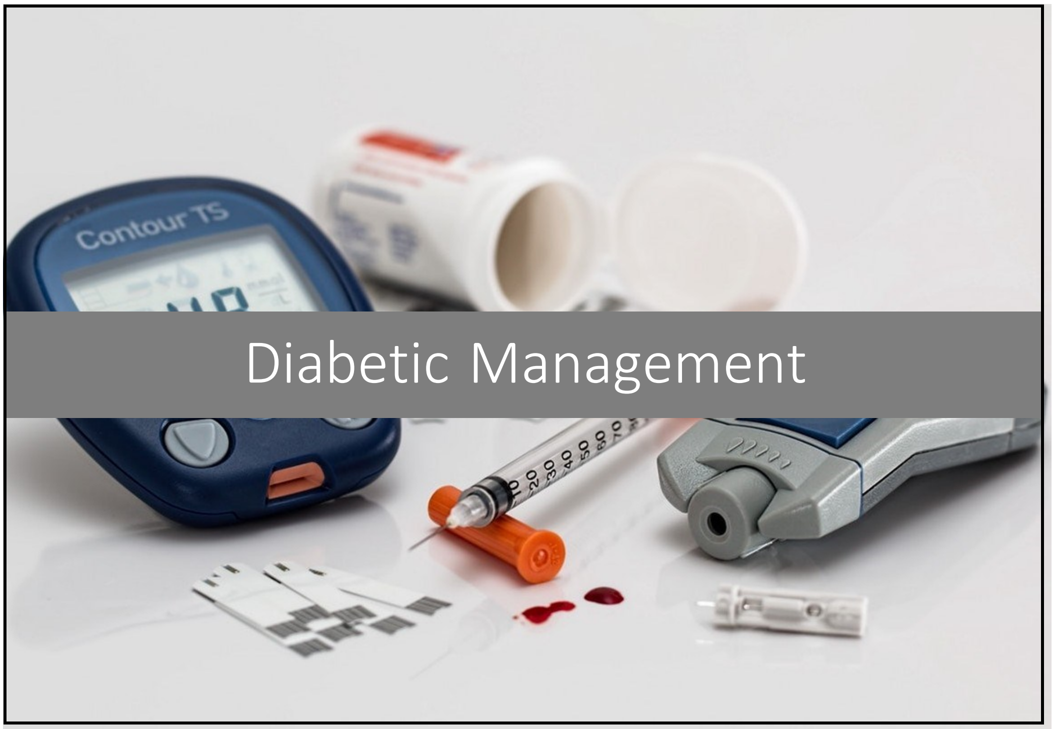

21

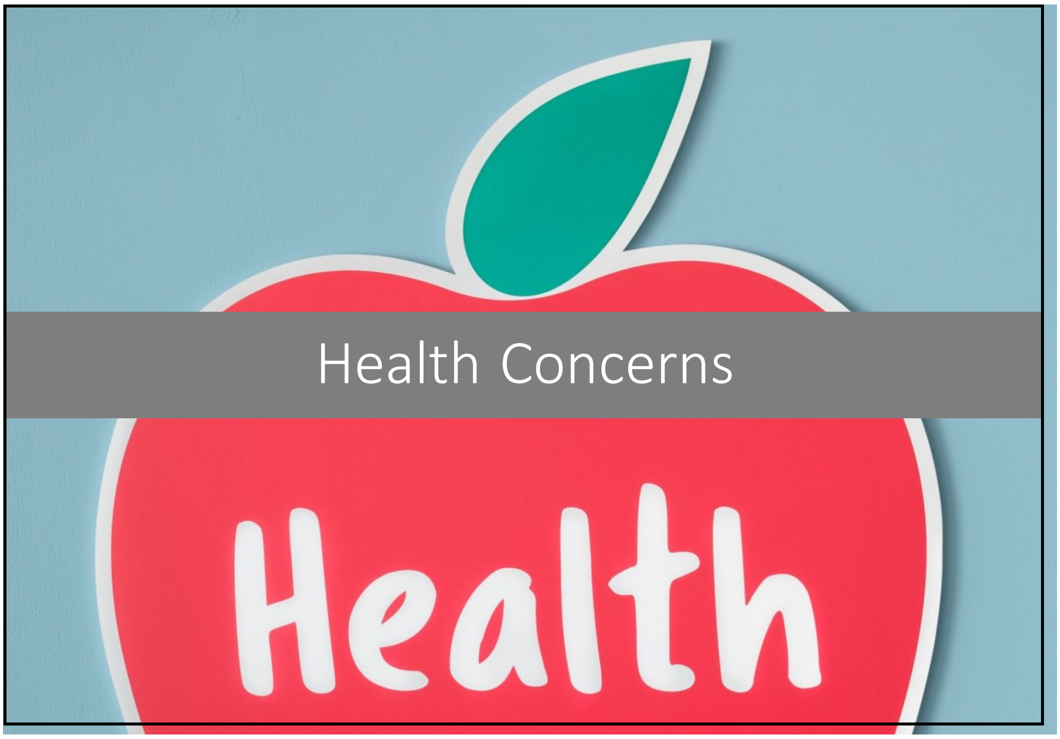

22

Diabetic Management

How do you mange your diabetes: diet, medication, sugar levels, exercise? Can you talk me through what you did yesterday?

When was the last time you got your eyes and kidney checked?

23

Health Concerns

Have you got any other health concerns?

24

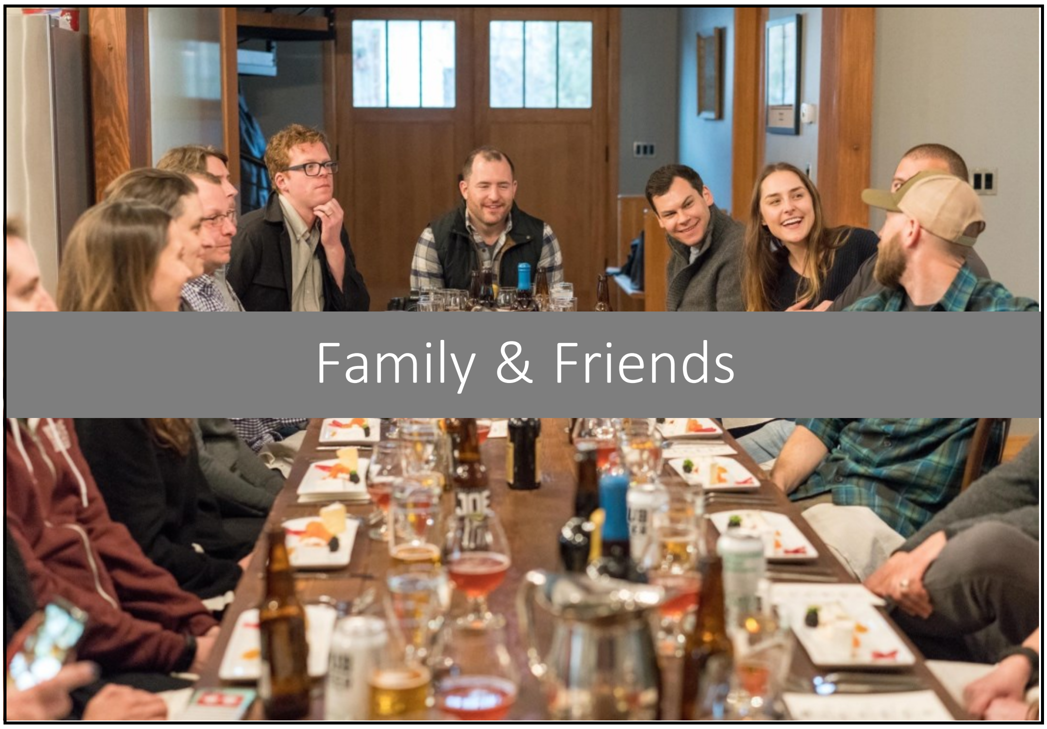

25

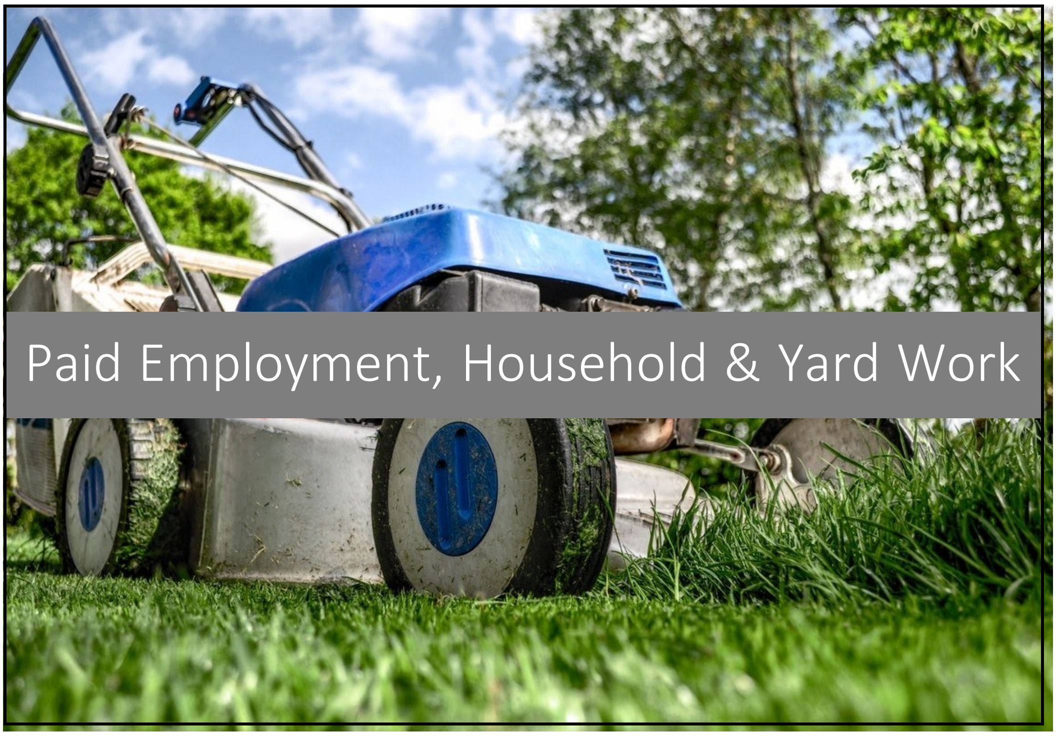

26

Family & Friends

Do you live on your own or with others?

Can you tell us more about the 3 most important people in your life?

How does the foot ulcer affect your relationship with your family or your friends?

27

Paid Employment, Household & Yard Work

Do you work?

How does the foot ulcer affect your work and your finances?

28

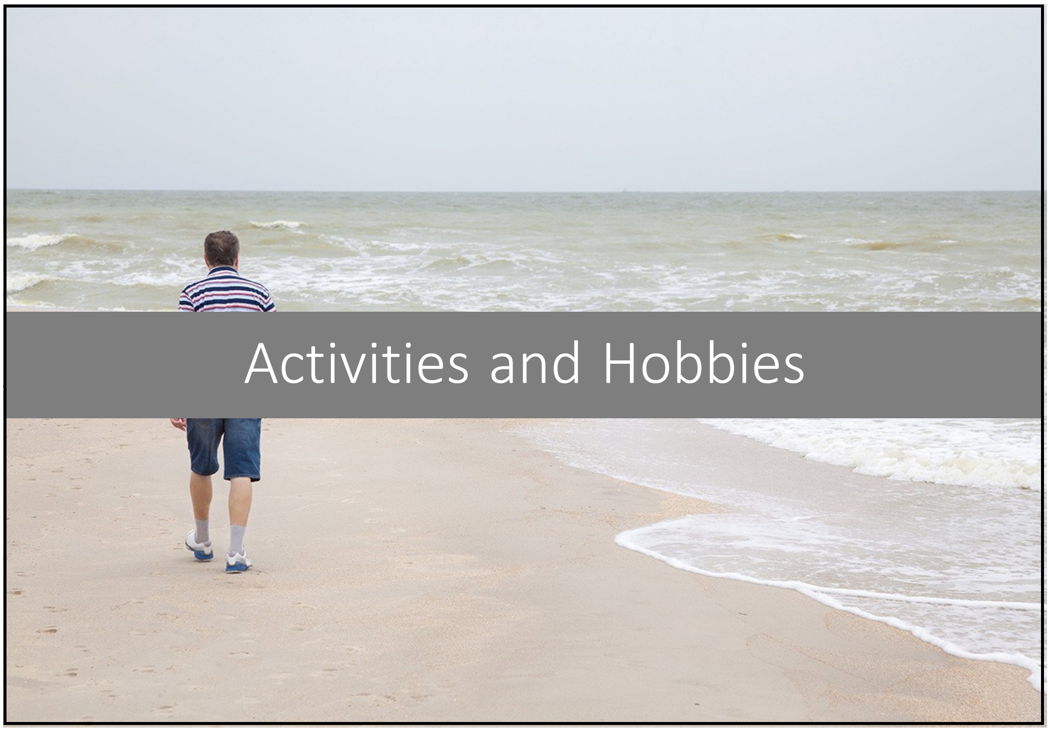

29

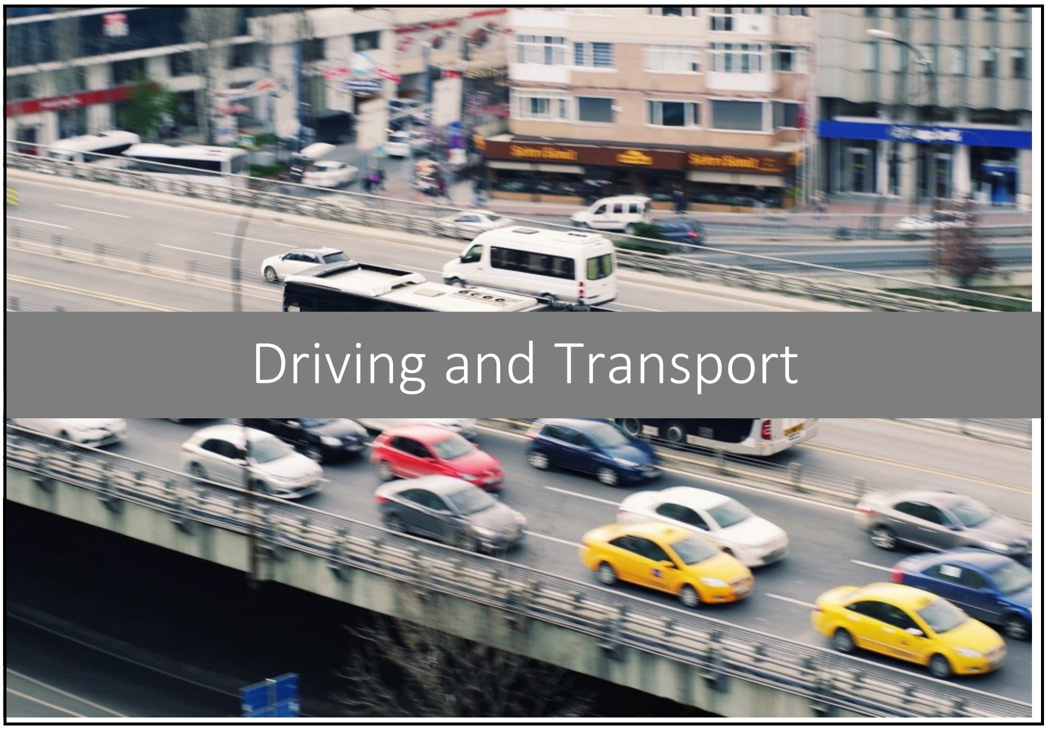

30

Activities and Hobbies

What do you like to do in your spare time?

How does the foot ulcer affect your spare time activities?

31

Driving and Transport

Do you drive a car / motorbike?

Do you use other forms of transport (public, ride a bike, walk)?

How does the foot ulcer affect driving and transportation?

32

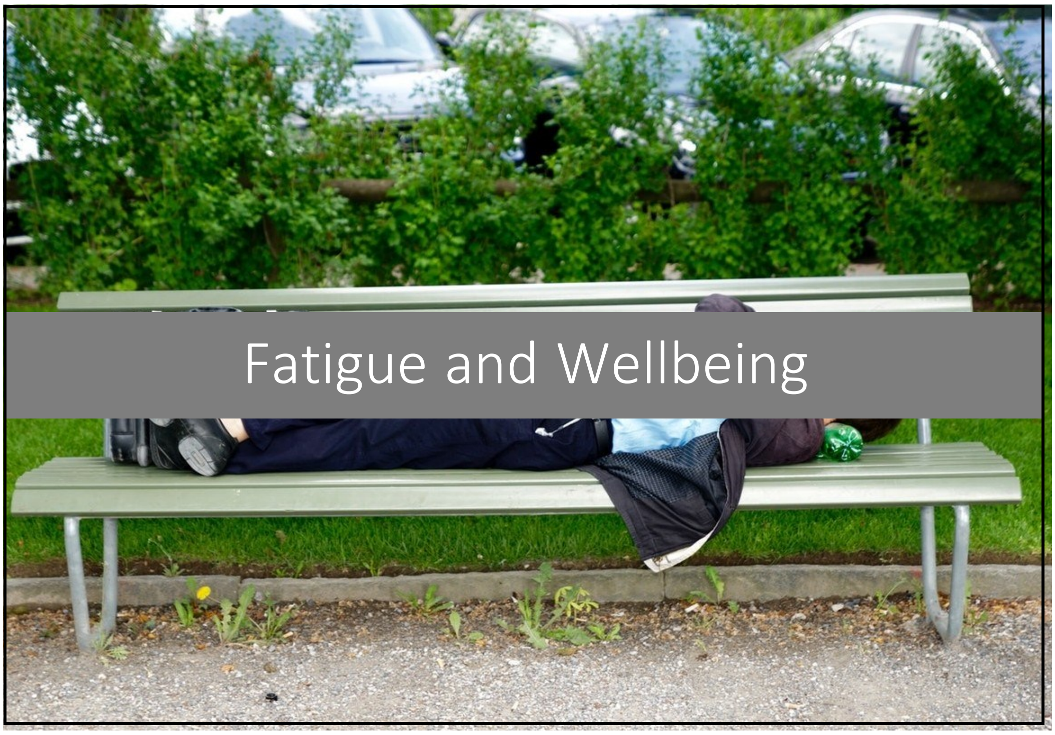

33

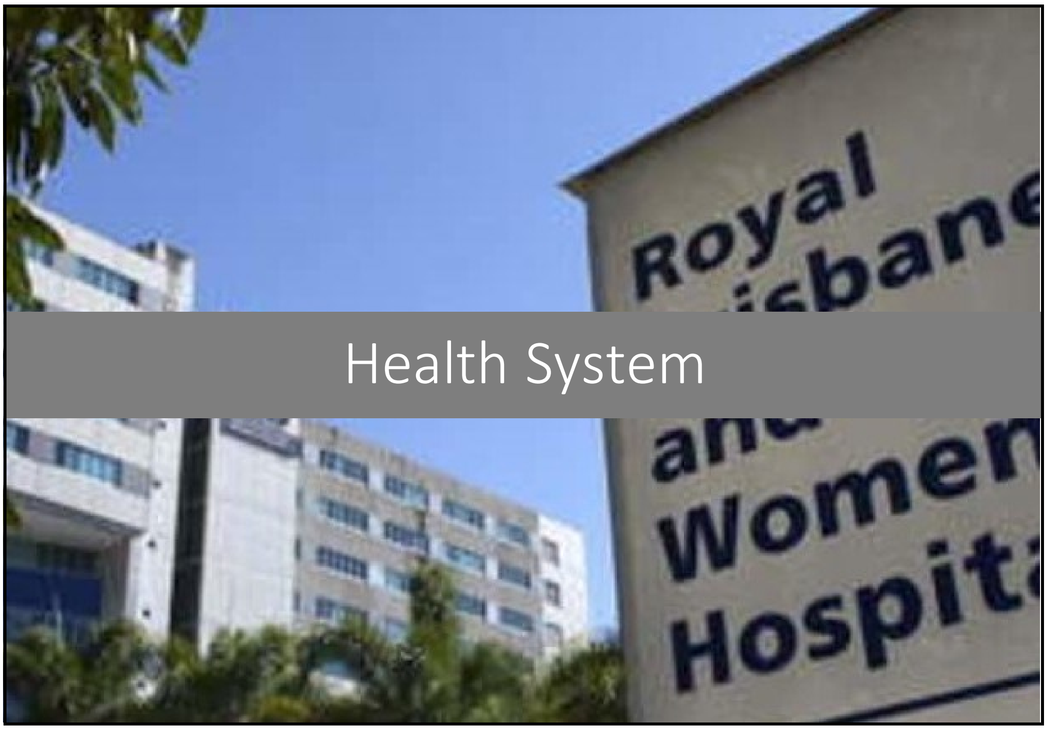

34

Fatigue and Wellbeing

How do you maintain your care over time? What are you struggling with? What keeps you going?

What do you to stay well? How do you keep yourself in a positive frame of mind?

35

Health System

What are the key services that you access?

What works well?

What could be improved?

36
